# PtrSAUR32 Interacts with PtrPP2C.Ds to Regulate Root Growth in Citrus

**DOI:** 10.3390/plants14111579

**Published:** 2025-05-22

**Authors:** Xiaoli Wang, Xiaoya Li, Saihang Zheng, Fusheng Wang, Shiping Zhu, Xiaochun Zhao

**Affiliations:** 1 Citrus Research Institute, Southwest University/Chinese Academy of Agricultural Sciences, Beibei, Chongqing 400712, China; 2 National Citrus Engineering Research Center, Beibei, Chongqing 400712, China; 3 Yibin Academy of Southwest University, Southwest University, Yibin 644005, China

**Keywords:** auxin, citrus, PtrSAUR32, PtrPP2C.Ds, root growth

## Abstract

Small auxin-up-regulated RNA (*SAUR*) genes are involved in the regulation of dynamic and adaptive growth in higher plants. However, their function and mode of action in citrus root growth are still unknown. Here, we demonstrate that in *Poncirus trifoliata*, *PtrSAUR32* acted downstream of the auxin response factor PtrARF8 to regulate root growth by interacting with PtrPP2C.Ds, subfamily type 2C protein phosphatases which interacted with H-ATPase and PtrHA. In this study, several members of *SAUR* family in *Poncirus trifoliata* are identified to be associated with the growth and development of the roots. Among them, *PtrSAUR32* was found to be highly expressed in the RT (root tip), and the level of its expression was significantly positively corelated to the length of primary roots (*p* < 0.01). The overexpression of *PtrSAUR32* in citrus significantly promoted the growth of primary roots. In *PtrSAUR32* transgenic citrus plants, the expressions of several auxin biosynthesis and transport genes were altered in accordance with the expression of *PtrSAUR32*. Y1H and dual-luciferase reporter assays proved that the expression of *PtrSUAR32* is regulated by PtrARF8. Y2H and BiFC assay results indicated that PtrSAUR32 interacted with PtrPP2C.Ds subfamily members PtrPP2C.D1, PtrPP2C.D6, and PtrPP2C.D7, of which PtrPP2C.D7 could interact with PtrHA in vivo.

## 1. Introduction

Plants are dependent on their roots to capture water and nutrients from the soil to maintain different kinds of complex life activities. Therefore, understanding the regulation of root system architecture (RSA) is of great significance to plant growth [1,2]. RSA, with a strong plasticity, is mainly determined by the morphological development of the roots, such as root depth, lateral root expansion, and root length densities [3]. RSA is regulated by both intrinsic (genetics) and environmental response pathways, and plant hormones such as auxin, cytokinin (CK), abscisic acid (ABA), brassinosteroid (BR), etc., play significant roles in the development of roots [4,5].

It is well known that auxin is a master regulator involved in almost every facet of plant root development [6,7,8]. In the auxin signaling mechanism, auxin response factors (ARFs) transcriptionally activate early auxin-responsive genes, such as auxin/indole-3-acetic acid (*Aux/IAA*), Gretchen Hagen3 (*GH3*), and small auxin-up RNA (*SAUR*) [9]. Normally, Aux/IAA proteins act as the negative regulators of auxin signaling by interacting with ARFs and *GH3* genes convert active IAA into its inactive form, while *SAURs* are primary auxin response genes [10,11,12]. In *Arabidopsis*, multiple AUXIN/IAA-ARF complexes participate in root development. For example, AtIAA28-AtARFs and AtSHY2/IAA3-AtARF modules cooperatively regulate the development of lateral roots [13,14]. Additionally, *AtGH3.3*, *AtGH3.5*, and *AtGH3.6* were required for adventitious root initiation [15]. All this evidence indicates that early auxin response genes play important roles in root development.

*SAURs*, the largest family of early auxin-responsive genes, are implicated in many biological processes, including leaf growth, senescence, and apical hook development, as well as root growth and development [11,16]. The best-known function of *SAURs* is to promote cell elongation [16,17]. For instance, a reduction in AtSAUR19-*24* expression led to diminished cell expansion [18]. Transgenic plants that express *AtSAUR63* promoted the elongation growth of hypocotyls, petals, and stamen filaments [19]. Root growth depends on spatially distinct cell division and elongation activities in the root meristem. The research shows that AtARF6/8 and AtARF7/19 can bind to the auxin response elements of *AtSAUR15* to regulate auxin-mediated lateral root and adventitious root formation [20]. In *Arabidopsis*, overexpressing *AtSAUR76* resulted in longer roots, indicating that *AtSAUR76* positively regulates the root growth [21]. *SAUR* is a large gene family with diversified functions. Transgenic plants overexpressing *OsSAUR39* had shorter primary roots and a significantly lower number of lateral roots, which means that some *SAURs* act as negative regulators in root development [22].

*SAURs* are a key factor in auxin-induced acid growth, in which auxin activates the plasma membrane (PM) H-ATPases, resulting in proton efflux, and finally changes the ability of cell wall expansion to drive cell expansion [17,23]. On the one hand, the expression of *SAUR* genes was rapidly up-regulated by auxin, and SAURs bonded to the proteins of phosphatase 2C D (PP2C.D) and inhibited their phosphatase activity [20,23,24,25]. On the other hand, PP2C.Ds physically interact with PM H-ATPases and negatively regulate their activity [17]. Together, SAUR interacts with PP2C.Ds to inhibit their activities, thereby stimulating the activities of plasma membrane H-ATPases, resulting in apoplastic acidification and PM hyperpolarization, and ultimately promoting an increase in cell size [17,23]. Meanwhile, some of *SAURs* like *AAM1* (*AtSAUR32*) and *AtSAUR36* seem to have an opposite role in cell expansion [26,27].

Citrus is one of the most important fruit crops worldwide. However, environmental stresses, such as drought, cold, and soil salinity, limit the productivity of citrus. The application of grafting techniques has largely enabled citrus to overcome these limitations, imparting desirable qualities on citrus such as yield improvement, enhanced growth, and stress resistance [28]. Therefore, the importance of rootstock in citrus production is self-evident. As the organs used by plants to perceive the soil environment, rootstock roots play crucial roles in responding to environmental stresses and directly affect the development and growth of a citrus plant. Even though the morphology of roots is important to the function of roots, the genetic regulation of formation of roots system is still largely unknown in citrus. The importance of *SAUR* genes in the regulation of dynamic and adaptive growth, and the molecular mechanisms of the action of SAUR proteins, have been revealed in recent years. However, most of these studies were conducted in *Arabidopsis* and other herbaceous plant species. The function and mode of action of *SAUR* genes in woody plants such as citrus plants still remain largely unknown. In our previous study, SAUR gene family members species were characterized in several citrus [29], and one SAUR gene was potentially related to the morphology of the root system by comparative transcriptomic analysis of the roots in different morphologies [30]. To understand the function of *SAURs* in citrus root growth and development, as well as the molecular mechanisms of root morphogenesis in citrus for improvement of the root system architecture and stress tolerance of citrus rootstocks, the function and mechanisms of *PtrSAUR32* in the regulation of citrus root growth and development were explored in this study.

## 2. Results

### 2.1. PtrSAUR Genes Related to Root Growth in Trifoliate Orange

**Figure 1:**
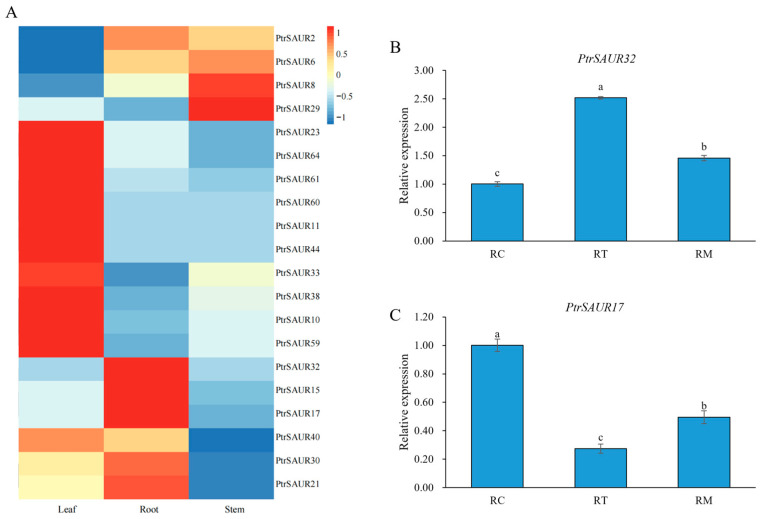
Characteristics of *PtrSAUR* genes expressions in citrus. (**A**) A heat map of *PtrSAURs* expression in different tissues. (**B**) Expression patterns of *PtrSAUR32* in different root zones. (**C**) Expression patterns of *PtrSAUR17* in different root zones. RT (root tip): the meristematic/elongation zone of the root; RM (middle part of root): root elongation/differentiation and lateral root initiation zone; RC (root collar): lateral root growth zone. Different letters (a–c) indicate the level of significant difference at *p* < 0.05.

In this study, trifoliate orange (*P. trifoliata*), one of the most important citrus rootstocks, was employed to study the functions of *SAUR* genes in the root growth and development of citrus. There were 68 *SAUR* genes identified from *P. trifoliata*. In this study, we chose 20 of them, which were homologues of *AtSAURs* involved in *Arabidopsis* root growth, for investigation of the relationship of *SAUR* with the growth of citrus roots. Transcriptional analysis of the *PtrSAUR* genes in the root, stem, and leaf of trifoliate orange demonstrated that the expression of some *PtrSAUR* genes was tissue-specific, among which *PtrSAUR32*, *PtrSAUR17*, and *PtrSAUR15* had significantly higher expressions in the root than in the stem and leaf, indicating that *PtrSAUR32*, *PtrSAUR17,* and *PtrSAUR15* might be involved in root development and growth in trifoliate orange (Figure 1A). Both *PtrSAUR32* and *PtrSAUR17* showed a more predominant tissue-specific expression in the root and were selected for further study. The expressions of *PtrSAUR32* and *PtrSAUR17* in different zones of the root were analyzed (Figure 1B,C). The expression of *PtrSAUR32* was higher in the meristematic/elongation zone (RT) and lower in the elongation/differentiation and lateral root initiation zone (RM) and lateral root growth zone (RC), while the expression of *PtrSAUR17* in the RC was higher than in the RT and RM, suggesting that *PtrSAUR32* and *PtrSAUR17* may play different roles in root morphogenesis.

### 2.2. SAUR32 Involved in Regulating Root Growth in Citrus

**Figure 2:**
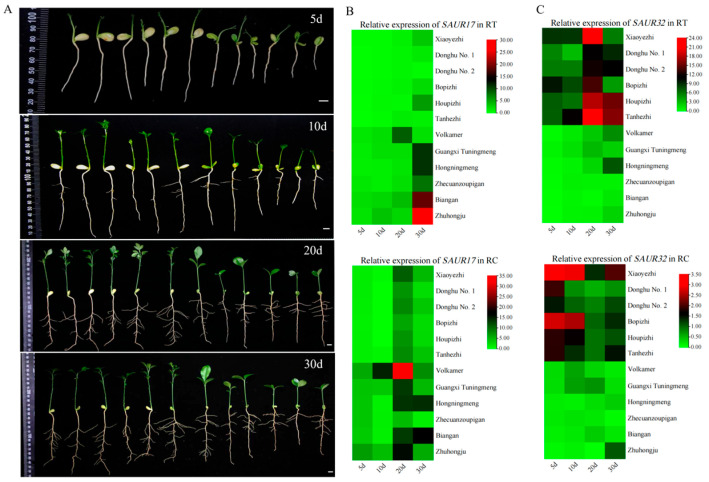
Root indexes and expressions of *SAUR17/32* in RT and RC. (**A**) Root morphology of citrus rootstocks at different days after sowing. From left to right are *Poncirus trifoliata* varieties (Xiaoyezhi, Donghu No. 1, Donghu No. 2, Bopizhi, Houpizhi, Tanhezhi), *C. volkameriana* variety (Volkamer), *C. limonia* variety (Guangxi Tuningmeng), *C. limon* variety (Hongningmeng), and *C*. *reticulata* varieties (Zhecuanzoupigan, Biangan, Zhuhongju). Bar = 1 cm. (**B**) Expression patterns of *SAUR17* in RT and RCs. (**C**) Expression patterns of *SAUR32* in RT and RCs.

Several species of citrus varieties are used as rootstocks in citrus production. The morphologies of their root systems are significantly different [30]. Here, relationship between *SAUR17* and *SAUR32* genes and root morphology was investigated with the seedlings of 12 citrus rootstock varieties differing in root morphology. The seedlings of *P. trifoliata* varieties had the longest primary roots at 30 days post sowing (dps), while the seedlings of *C. reticulata* varieties showed the shortest length of primary roots. Most of *P. trifoliata* varieties (except Donghu No. 1) and *Volkamer* produced more lateral roots than other varieties (Figure 2A, Supplementary Table S3). At 10–20 dps, the lateral roots rapidly generated from the primary root (Figure 2A, Table S3).

**Figure 3:**
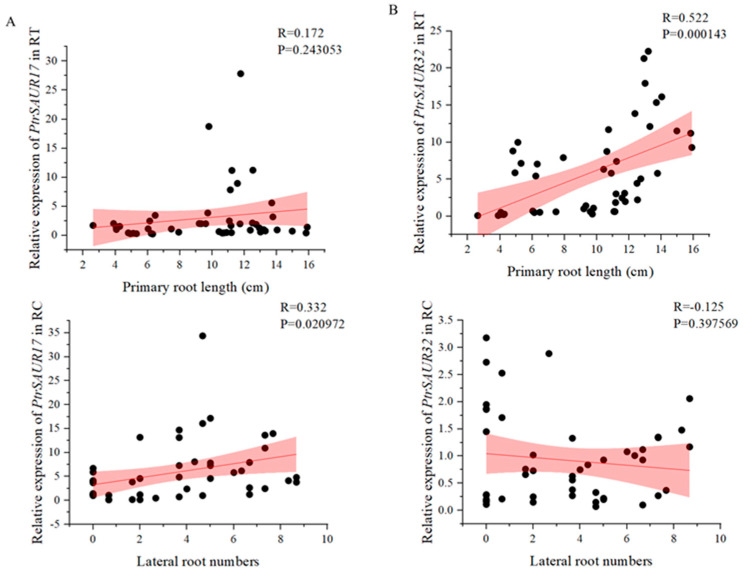
Correlation analysis between root indexes and the expression of *PtrSAUR17/32*. (**A**) Relationship between root indexes and the expression of *PtrSAUR17*. (**B**) Relationship between root indexes and the expression of *PtrSAUR32*.

The expressions of two *SAUR* genes demonstrated different associations with rootstock varieties and the development of roots. *SAUR32* showed more distinct expression in the varieties with longer primary roots, such as *P. trifoliata* varieties, than that of *SAUR17* (Figure 2B,C). High expression of *SAUR32* was observed in the RC at 5–10 dps, and in the RT at 20–30 dps in *P. trifoliata* varieties. The high activity of *SAUR17* was only observed in the RT at 30 dps of Zhuhongju, and the RC at 20 dps of Volkamer and Biangan. The results of the correlation analysis indicate that the expression level of *SAUR32* in the RT was extremely significantly correlated to the length of primary roots (*p* < 0.01), and the expression of *SAUR17* in the RC was significantly correlated to the number of lateral roots (*p* < 0.05) (Figure 3A,B). However, the level of *SAUR17* expression did not show an obvious correlation with the length of primary roots. Therefore, we did not consider it as a candidate gene for the further investigation of its function in root growth. The expression of *SAUR32* showed an extremely significant correlation with the growth of primary roots, and a positive association with the varieties with longer roots; therefore, it was selected as a major candidate gene for further functional study of its role in root growth.

### 2.3. Molecular Characteristcs of PtrSAUR32

**Figure 4:**
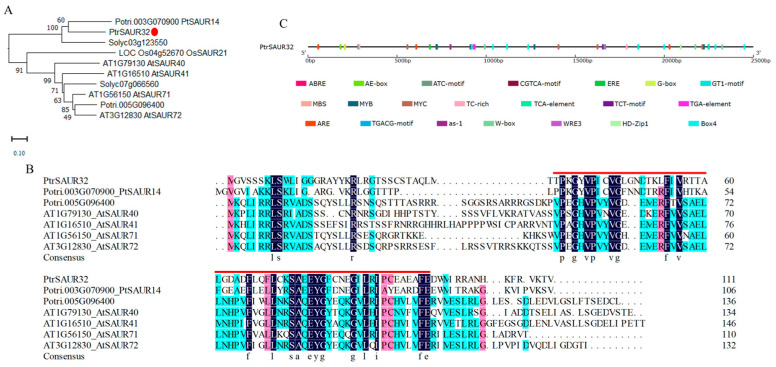
Bioinformatic analysis of PtrSAUR32. (**A**) Phylogenic tree of *PtrSAUR32* and its homologues. The homologues are from *Arabidopsis thaliana*, *Oryza sativa*, *Solanum lycopersicum,* and *Populus* L. *PtrSAUR32* is indicated with a red dot. (**B**) Multiple-sequence alignment of PtrSAUR32 with *Arabidopsis* and poplar homologous. The SAUR-specific domain is highlighted with a red line. (**C**) The *cis*-acting elements in the promoter of *PtrSAUR32*.

*PtrSAUR32* encodes a protein of 111 amino acid residues with a molecular weight of 12.35 kDa and theoretical pI of 9.1. A phylogenetic tree constructed with *PtrSAUR32* and its homologous genes from several species showed that *PtrSAUR32* is homologous to *PtSAUR14* of poplar, *Solyc03g123550* of tomato, and *AtSAUR40/41* and *AtSAUR71/72* of *A. thaliana* (Figure 4A), but it only shares low similarities in its amino acid sequence: 23.53%, 20.13%, 24.78% and 24.63%, respectively. Multiple-sequence comparative analysis revealed that PtrSAUR32 contains a SAUR-specific domain SSD (Figure 4B), which is highly conserved [16,31,32]. Figure 4C shows that there are many predicted *cis*-acting elements located in the promoter of *PtrSAUR32*, such as light-responsive elements’ AE-box, ATC-motif, Box4, etc.; hormone response elements’ ABRE, CGTCA-motif, TGA-element, etc.; stress response elements’ MBS and TC-rich repeats; the element essential for anaerobic induction ARE; and the element involved in the differentiation of the palisade mesophyll cells HD-Zip1. All of this suggests that the expression of *PtrSAUR32* may be regulated by various factors, such as light, plant hormones, and stresses.

### 2.4. Reponse of PtrSAUR32 to Auxin and Abiotic Stresses

**Figure 5:**
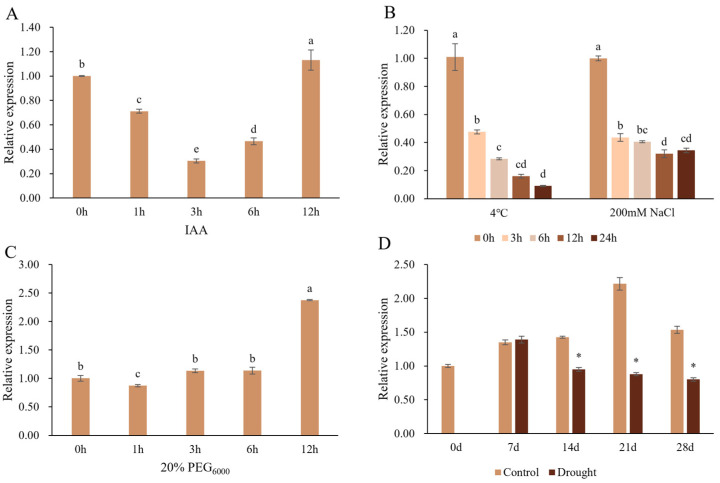
The expression analysis of *PtrSAUR32* under abiotic stress and IAA treatments. (**A**) IAA treatment. (**B**) Cold and NaCl treatments. (**C**) PEG treatment. (**D**) Drought treatment. Different letters (a–e) and asterisks (*) mean significant difference at *p* < 0.05.

To explore whether the expression of *PtrSAUR32* can be induced by auxin and abiotic stresses, the relative expression of *PtrSAUR32* under various treatments was analyzed with RT-qPCR. The results show that the level of *PtrSAUR32* expression was first reduced and then increased under IAA treatment (Figure 5A). Under cold and salt treatments, the expression of *PtrSAUR32* decreased (Figure 5B), while under PEG treatment, the expression of *PtrSAUR32* increased (Figure 5C). Drought treatment slightly promoted the expression of *PtrSAUR32* in the first 7 days and then significantly suppressed its expression in the rest period time of treatment (Figure 5D). These results indicate that the transcription of *PtrSAUR32* is affected by auxin and abiotic stresses.

### 2.5. PtrSAUR32 Promotes the Growth and Development of Root

**Figure 6:**
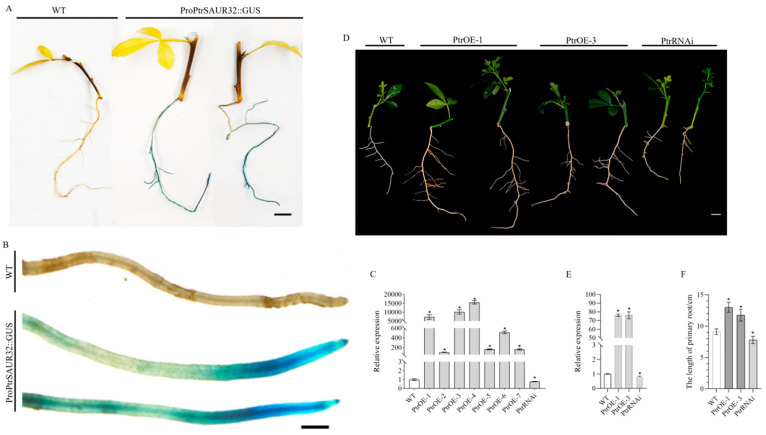
Functional analysis of *PtrSAUR32* in citrus. (**A**) GUS staining of *PtrSAUR32::GUS* transgenic citrus. Bar = 1 cm. (**B**) GUS staining of root tip. Bar = 1 mm. (**C**) The expression of *PtrSAUR32* in the leaves of transgenic citrus. (**D**) Morphological characteristics of transgenic citrus roots. Bar = 1 cm. (**E**) The expression of *PtrSAUR32* in the roots of transgenic citrus. (**F**) Statistical analysis of primary roots length. * means significant difference at *p* <  0.05.

To further analyze the expression patterns of *PtrSAUR32*, we obtained the *ProPtrSAUR32::GUS* transgenic plants. *PtrSAUR32* was mainly expressed in the roots, and a strong GUS activity was observed in the root tips (Figure 6A,B). The function of *PtrSAUR32* in regulating the root growth and development of citrus root was verified with transgenic trifoliate orange plants. The plants with self roots were generated from the cuttings of two *PtrSAUR32* overexpression plants (PtrOE-1, PtrOE-3) and one silenced plant (PtrRNAi) (Figure 6C). The length of primary roots was significantly increased and decreased by the overexpression and silencing of *PtrSAUR32*, respectively, which shows a significant correlation with the expression level of *PtrSAUR32* in the roots of transgenic plants (Figure 6D–F). These results suggest that *PtrSAUR32* plays an important role in regulating the growth and development of roots in citrus.

### 2.6. PtrSAUR32 Interacts with the Genes Related to the Growth and Development of Root

**Figure 7:**
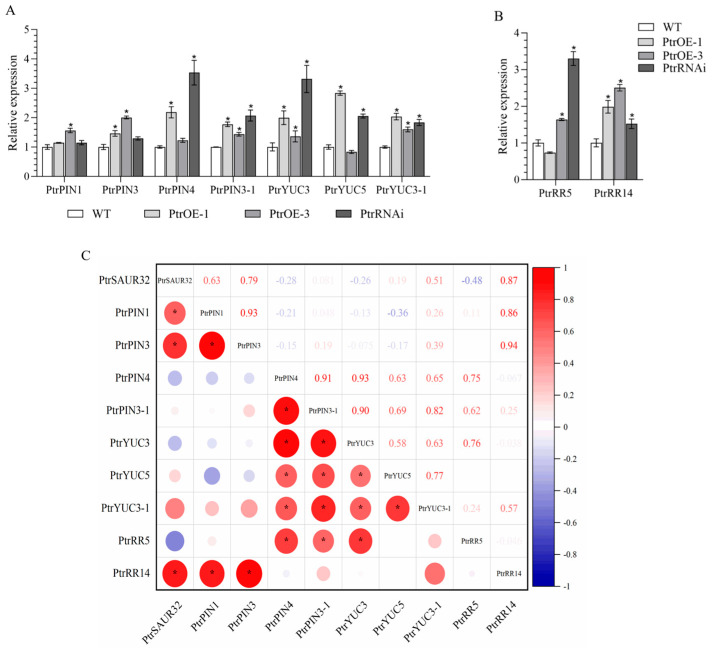
The expression levels of *PtrYUCs*, *PtrPINs*, and *PtrRRs* in transgenic citrus plants roots. (**A**) The expression of *PtrYUCs* and *PtrPINs* in roots. (**B**) The expression of *PtrRRs* in roots. (**C**) The correlation between expressions of *PtrSAUR32* and related genes in the roots. * means significant difference at *p* < 0.05.

To explore the mechanism of *PtrSAUR32* regulation in root growth, the association between *PtrSAUR32* and genes involved in auxin and cytokinin signal pathways related to root growth and root development was investigated. The results demonstrate that in *PtrSAUR32* transgenic plants, the expression of response regulator (*RR*) *PtrRR14* significantly corresponded with the expression of *PtrSAUR32* (Figure 7B,C). The expression of both *PtrYUCs* and *PtrPINs* were influenced by *PtrSAUR32* (Figure 7A). Compared to WT, the expressions of *PtrPINs* and *PtrYUCs* were higher in both the overexpressing and silenced plants. Among them, *PtrPIN1* and *PtrPIN3* showed significant positive correlations with *PtrSAUR32*, while the negative correlation of *PtrPIN4*, *PtrPIN3-1*, and *PtrYUC3* with *PtrSAUR32*, and the positive correlation of *PtrYUC5* and *PtrYUC3-1* with *PtrSAUR32* were not significant (Figure 7C).

**Figure 8:**
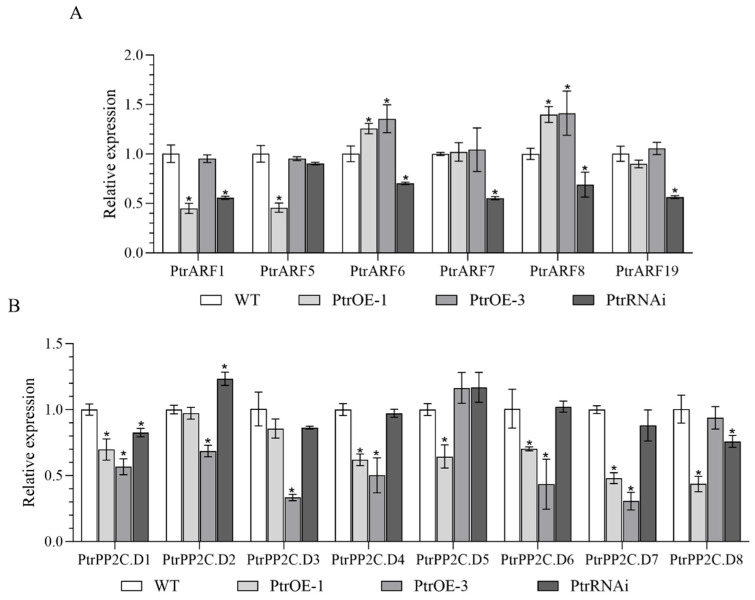
The expression levels of *PtrARFs* and *PtrPP2C.Ds* in transgenic citrus plants roots. (**A**) The expression of *PtrARFs* in roots. (**B**) The expression of *PtrPP2C.Ds* in roots. * means significant difference at *p* < 0.05.

In *P. trifoliata*, six members of *A-ARFs*, termed as *PtrARF1*, *PtrARF5*, *PtrARF6*, *PtrARF7*, *PtrARF8,* and *PtrARF19* in this study, were identified to be homologous to *A-ARFs* of *Arabidopsis* (Figure S1), which are closely related to root organogenesis in *Arabidopsis* [33]. In PtrSAUR32 transgenic plants, the expressions of *PtrARF6* and *PtrARF8* were significantly correlated to the expression of *PtrSAUR32* (Figure 8A). Root growth is the consequence of cell divisions and cell expansion, of which SAURs bond to the PP2C.D to intervene in the interaction of PP2C.D with AHA to drive cell expansion [17,23]. In this study, the expression of eight *PtrPP2C.D* genes (termed as *PtrPP2C.D1*, *PtrPP2C.D2*, *PtrPP2C.D3*, *PtrPP2C.D4*, *PtrPP2C.D5*, *PtrPP2C.D6*, *PtrPP2C.D7* and *PtrPP2C.D8* for *PtrPP2C30*, *PtrPP2C41*, *PtrPP2C35*, *PtrPP2C18*, *PtrPP2C38*, *PtrPP2C36*, *PtrPP2C37,* and *PtrPP2C27,* as reported by Yang et al. [34], respectively) were analyzed in transgenic plants. Overall, the expressions of *PtrPP2C.Ds* were down-regulated in the *PtrSAUR32* overexpression transgenic lines, and up-regulated in the *PtrSAUR32* silenced transgenic plant (Figure 8B). This indicates that *PtrSAUR32* negatively affects the expression of *PtrPP2C.Ds.* Taken together, the above results suggest that *PtrSAUR32* may regulate root growth and development in citrus through interaction with *PtrARFs* and *PtrPP2C.Ds*.

### 2.7. PtrSAUR32 Interacts with PtrPP2C.Ds

**Figure 9:**
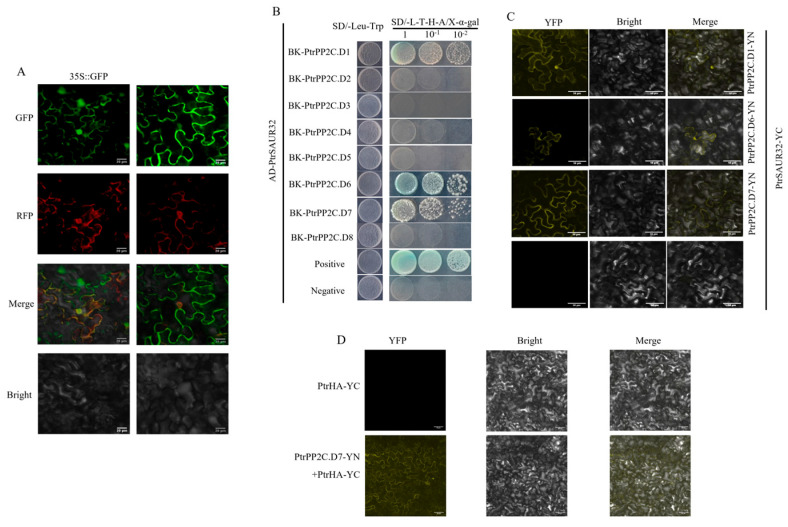
PtrSAUR32 interacted with PtrPP2C.Ds. (**A**) Subcellular localization of PtrSAUR32. Bars = 20 μm. (**B**) Interaction assay between PtrSAUR32 and PtrPP2CDs via yeast two-hybrid system. SD/-Leu-Trp, SD medium without Leu and Trp; SD/-L-T-H-A/X-α-gal, SD medium without Leu, Trp, His and Ade but containing X-α-gal (40 µg/mL). Positive: pGADT7-T and pGBKT7-53. Negative: pGADT7-T and pGBKT7-Lam. (**C**) BiFC analysis of the interaction between PtrSAUR32 and PtrPP2C.Ds. Bars = 50 μm. (**D**) The physical interaction between PtrPP2C.D7 and PtrHA. Bars = 50 μm.

The above results reveal the positive relationship between *PtrSAUR32* and *PtrPP2C.Ds* in citrus. It can be presumed that PtrSAUR32 may interact with PtrPP2C.Ds as in *Arabidopsis* [25]. To this end, we first analyzed the subcellular localization of PtrSAUR32. A transient expression assay in *N. benthamiana* leaves showed that a strong GFP fluorescent signal tagged to PtrSAUR32 was observed at the PM (plasma membrane) (Figure 9A), suggesting that PtrSAUR32 is located at the PM and can interact with PtrPP2C.Ds. The yeast two-hybrid assay (Y2H) showed that the yeast cells could grow normally and the color of colonies turned to blue on SD/-Leu-Trp-His-Ade (SD/-L-T-H-A) medium supplemented with X-α-gal when PtrSAUR32 was co-transformed with PtrPP2C.D1, PtrPP2C.D6, and PtrPP2C.D7, respectively (Figure 9B), indicating that PtrSAUR32 could interact with PtrPP2C.D1, PtrPP2C.D6, and PtrPP2C.D7. A further bimolecular fluorescence complementation experiment (BiFC) confirmed the interactions of PtrSAUR32 with PtrPP2C.D1, PtrPP2C.D6, and PtrPP2C.D7 at the PM (Figure 9C). These results suggest that *PtrSAUR32* may be a member of the SAURs in citrus that interacts with PP2C.D to regulate the activity of H-ATPase (HA) and affect the cell expansion.

Multiple comparative analyses revealed that the amino acids sequence of Ptrif.0001s2325 (termed as PtrHA) has high similarities of 87% and 85% with AHA1 and AHA2, respectively (Figure S2), suggesting that PtrHA may also play the same role as AHA1 and AHA2. The BiFC assay results show that a strong YFP fluorescent signal was observed at the plasma membrane when PtrHA and PtrPP2C.D7 were co-injected into tobacco leaves, indicating that PtrPP2C.D7 interacted with PtrHA (Figure 9D).

### 2.8. PtrSAUR32 Is the Direct Target of PtrARF8

**Figure 10:**
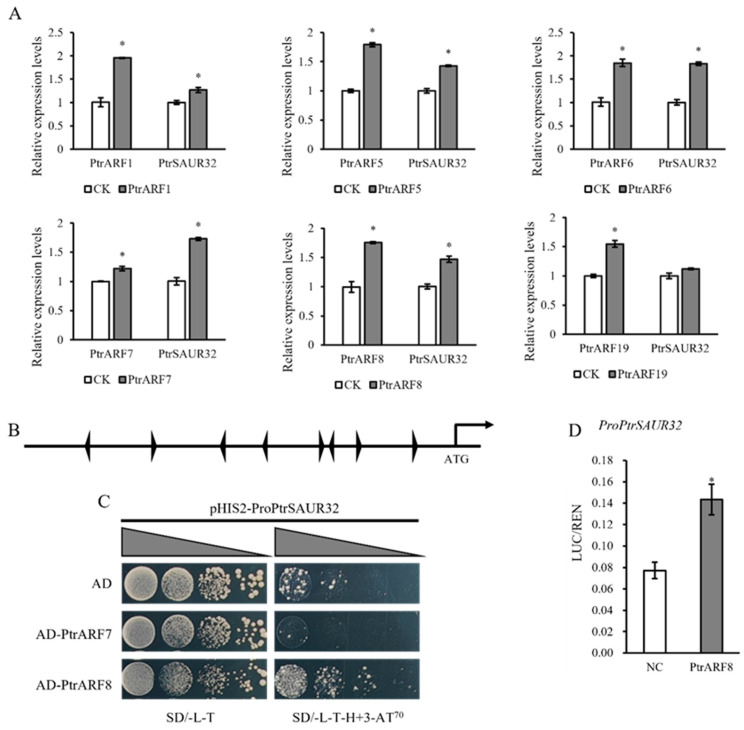
The expression of *PtrSAUR32* was activated by PtrARF8. (**A**) Expression analysis of *PtrSAUR32* in citrus leaves transiently expressing *PtrAFRs*. (**B**) Identification of auxin response elements (AuxREs) in *PtrSAUR32* promoter. (**C**) Yeast one-hybrid (Y1H) assay showed the interaction between *PtrSAUR32* promoter and PtrARFs. The yeast cells were diluted with 0.9% NaCl into four concentration gradients (10^−1^, 10^−2^, 10^−3^ and 10^−4^) and then cultured in SD/-L/-T medium and SD/-L-T-H medium containing 70 mM 3-AT. SD/-L-T, SD medium without Leu and Trp; SD/-L-T-H, SD medium without Leu, Trp, and His. (**D**) Dual-luciferase reporter assay showing that PtrARF8 activates the transcription level of *PtrSAUR32*. NC: Negative Control. * means significant difference at *p* < 0.05.

In auxin-mediated acid growth theory, the expression of *SAUR* genes is regulated through the canonical SCF^TIR1/AFB^ signaling pathway, in which the auxin response factors, ARFs, play an important role; additionally, *AtSAUR15* acts downstream of *AtARFs* to regulate auxin signaling-mediated root formation [20,23]. In *PtrSAUR32* transgenic citrus plants, the expression of *PtrARFs* demonstrated a strong correlation with *PtrSAUR32*, suggesting that PtrARFs possibly interact with *PtrSAUR32*. To verify this assumption, the CDS of *PtrARF1*, *PtrARF5*, *PtrARF6*, *PtrARF7*, *PtrARF8*, and *PtrARF19* were transiently expressed, respectively, in citrus leaves for investigation of the expression of *PtrSAUR32*. The results show that compared to the control, the expression of *PtrSAUR32* was significantly up-regulated by the transient overexpression of *PtrARF1*, *PtrARF5*, *PtrARF6*, *PtrARF7*, and *PtrARF8*, but not by *PtrARF19* (Figure 10A). The above results suggest that the expression of *PtrSAUR32* might be regulated by *PtrARFs*.

The bioinformatic analysis indicates that eight auxin-responsive elements (AuxREs) are located in the promoter region of *PtrSAUR32* (Figure 10B). According to the results of the qPCR analysis, PtrARF6, PtrARF7, and PtrARF8 were selected for determining their interaction with *PtrSAUR32*. The coding sequences of *PtrARF6*, *PtrARF7,* and *PtrARF8* were constructed into the empty vector PGADT7, and the *PtrSAUR32* promoter was fused to the empty vector pHIS2 to perform a yeast-one-hybrid (Y1H) assay to detect the bindings between PtrARFs and the *PtrSAUR32* promoter. PtrARF6 was not considered in the subsequent experiments due to yeast cells containing both the transcription factor PtrARF6 and the promoter of PtrSAUR32. The Y1H assay showed that the yeast cells grew better on the SD/-L-T-H medium containing 70 mM 3-AT when *ProPtrSAUR32* co-transformed with PtrARF8, indicating that PtrARF8 was able to bind to the promoter of *PtrSAUR32* in vitro (Figure 10C). This interaction was further confirmed by the dual-luciferase reporter assay. A higher ration of LUC/REN was detected in the leaves of tobacco plants transiently expressing PtrARF8 and *ProPtrSAUR32* constructs than those of NC (Figure 10D). These results demonstrate that PtrARF8 is the transcription factor of *PtrSAUR32*.

## 3. Discussion

### 3.1. PtrSAUR32 Is an Important Gene in Regulating Citrus Root Growth

Auxin plays a critical role in root morphogenesis [8]. The early auxin-responsive gene family *SAUR* is involved in multiple processes mediated by auxin, such as hypocotyl growth, apical hook development, and cotyledon open [18,25,35], and it participates in the regulation of root growth and development [20,21]. This study reveals that the transcription of *PtrSAUR32* was significantly related to the growth of primary roots in *P. trifoliata*. The overexpression and silencing of *PtrSAUR32* confirmed that *PtrSAUR32* is an important gene regulating the growth and development of roots in citrus, which is in agreement with the findings of Qiu et al. in *Arabidopsis* [36]. Additionally, the homologues of *PtrSAUR32* in *Arabidopsis (AtSAUR40/41/71/72)* could modulate ion homeostasis and salt tolerance [36]. It is worth to carrying out further experiments to verify whether *PtrSAUR32* can also improve the abiotic stress tolerance of citrus. Root development is a fairly complex process regulated by the interaction of auxin with other plant hormones. It is well known that cytokinin crosstalk with auxin controls root meristem size and ensures root growth [37,38,39]. Further, 10^−8^ to 10^−4^ M BA affected the elongation of *Arabidopsis* roots [40]. In this study, the expression of the cytokinin response regulator *PtrRR14* significantly corresponded to *PtrASUR32* in transgenic citrus plants, providing further evidence to prove that *PtrSAUR32* may be involved in the cytokinin signaling-mediated root growth and development.

### 3.2. The Mechanism of PtrSAUR32 Regulation in Root Growth and Development in Citrus

In *Arabidopsis*, AtSAUR15 interacts with AtPP2C.Ds to inhibit their activities, thereby stimulating plasma membrane H-ATPases, which drive cell expansion and facilitate LR and AR formation [20]. In *P. trifoliata*, PtrSAUR32 also interacts with three PtrPP2C.D proteins (PtrPP2C.D1, PtrPP2C.D6, and PtrPP2C.D7), of which PtrPP2C.D7 can bind with PtrHA in the plasma membrane in vivo, indicating that PtrPP2C.Ds could regulate the activity of PM H-ATPase in citrus. In *Arabidopsis*, three AUTOINHIBITED PLASMA MEMBRANE H^+^-ATPases *AHA1*, *AHA2,* and *AHA7* are predominant in root epidermal cells, in which *AHA2* drives root cell expansion during growth [41]. *PtrHA* is highly homologous to *AHA2*, and it interacts with PtrPP2C.D7; therefore, it can be assumed that *PtrHA* plays a similar role in citrus root development to *AHA2* in *Arabidopsis*.

It had been proved that the promotion of root organogenesis in *Arabidopsis* by *SAUR15* is regulated by ARF6 and ARF7 [20]. Our study demonstrates that *PtrSAUR32* is regulated by PtrARF8. As an important hormone, auxin biosynthesis and transport and auxin-dependent signaling processes all affect root development [42,43]. *YUCCA* (*YUC*) genes are known as the key rate-limiting enzymes that function in tryptophan-dependent auxin biosynthesis [44]. In *Arabidopsis*, *AtYUC3*, *AtYUC5*, *AtYUC7*, *AtYUC8,* and *AtYUC9* were highly expressed in the root to promote root development [45]. *PtrYUC3*, *PtrYUC5,* and *PtrYUC3-1* were homologues of *AtYUC3*, *AtYUC5*, *AtYUC7*, *AtYUC8,* and *AtYUC9*. However, the overexpression and silence of *PtrSAUR32* did not significantly affect the expressions of 3 *PtrYUCs* in this study. Further work is required to clarify whether the *PtrSAUR32* is directly involved in the regulation of auxin synthesis or if it interacts with other members of *PtrYUCs*. Auxin transport is essential for the establishment and maintenance of local auxin concentrations, and the auxin efflux carrier PIN proteins are the most crucial components of auxin transport [46,47]. In roots, *AtPIN1* was predominantly expressed in the stele and endodermis cells and was regulated the flow of auxin to the quiescent center (QC) in the root meristem, while *AtPIN3* and *AtPIN4* mediated the redistribution of auxin in the roots as well [48,49]. We detected the expression levels of homologous genes *(PtrPIN1*, *PtrPIN3*, *PtrPIN4*, and *PtrPIN3-1)* of *AtPIN1, AtPIN3,* and *AtPIN4.* The expressions of *PtrPIN1* and *PtrPIN3* in *PtrSAUR32* transgenic plants were significantly increased, implying that *PtrSAUR32* might mediate the auxin transport in the roots of citrus.

### 3.3. Function of PtrSAUR32 in Response to Abiotic Stress

Bioinformatic analysis revealed a large number of stress-responsive *cis*-regulatory elements existing in the promoter of *PtrSAUR32*. The expression of *PtrSAUR32* under various abiotic stress treatments showed that *PtrSAUR32* significantly responded to the abiotic stresses, suggesting that *PtrSAUR32* may play a role in abiotic stress tolerance in citrus. The overexpression of *PavSAUR55* in *Arabidopsis* improved root elongation and showed higher tolerances to NaCl and mannitol treatments [50]. In this study, the expression of *PtrSAUR32* was up-regulated by osmatic and drought stresses, implying that *PtrSAUR32* may promote the adaptive growth of citrus roots in response to abiotic stress. This would possibly improve the tolerance of citrus to various abiotic stress such as drought. However, further experiments are required to clarify this presumption.

## 4. Materials and Methods

### 4.1. Plant Materials

Seven *Poncirus trifoliata* varieties (Donghu No. 1, Donghu No. 2, Houpizhi, Bopizhi, Tanhezhi, Xiaoyezhi, Donghaizhi), three *Citrus reticulata* varieties (Biangan, Zhecuanzoupigan, Zhuhongju), one *C. limon* variety (Hongningmeng), one *C. volkameriana* variety (Volkamer), and one *C. limonia* variety (Guangxi Tuningmeng) were used as plant materials in this study. All citrus varieties were provided by the National Citrus Germplasm Repository (Chongqing, China). Donghaizhi was used for the RT–qPCR analysis of gene expressions in different tissues and organs, under different hormone treatments and stresses, and under stable genetic transformation. Other varieties were used for analyzing the correlation between root indexes and gene expression in the roots of seedlings at different developmental stages. Further, 30-day-old tobacco (*Nicotiana benthamiana*) plants were used for the transient expression of candidate genes. All citrus and tobacco plants were grown in a greenhouse under 26 °C and a 16 h light/8 h dark cycle.

### 4.2. Treatments

Citrus seeds were germinated at 28 °C on wet filter paper in a Petri dish after removal of the seed coat and then cultured in pots filled with vermiculite under 26 °C and a 16 h light/8 h dark cycle, 12000LX for 30 days; roots, stems, and leaves were subsequently collected for analysis of the expression of *PtrSAURs*. Additionally, the root was divided into different zones for gene expression analysis, termed according to Zhang et al. [51] as the RT (root tip): the meristematic/elongation zone; RM (middle part of root): the root elongation/differentiation and lateral root initiation zone; and RC (Root collar): lateral root growth zone. In order to analyze the correlation between gene expression and root development, different zones of the root were sampled at 5, 10, 20, and 30 days after sowing; at the same time, the number of lateral roots in the RC and the length of the primary root were determined.

After citrus seedlings were grown under 26 °C and a 16 h light/8 h dark cycle for 30 days, abiotic stress and hormone treatments were carried out. Root samples were collected for qPCR analysis after treatment with 1µM IAA and 20% PEG6000 for 0, 1, 3, 6, 12 h, respectively. Root samples were collected at 0, 3, 6, 12, and 24 h after salt stress (200 mM NaCl) and cold treatments (4 °C). For drought treatment, the water supply was withheld after 30 days of normal conditions, and root samples were taken at 7, 14, 21, and 28 days, respectively.

### 4.3. DNA, RNA Extraction, and Gene Expression Analysis

Genomic DNA was extracted according CTAB Plant DNA Extraction Kit (Biomed, Beijing, China). Total RNA was extracted using the EASYspin Plus Plant RNA Kit (Aidlab, Beijing, China), and first-strand cDNA synthesis was performed using RevertAid™ Master Mix (Thermo Scientific, Waltham, MA, USA). First-strand cDNA was used as a template for RT–qPCR to analyze gene expression, as described in previous study [51]. The primer sequences used in this study are listed in Supplementary Data Table S1.

### 4.4. Bioinformatic Analysis

The website Expasy (https://www.expasy.org/resources/protparam) (accessed on 29 March 2023) was used to compute the physical and chemical parameters of PtrSAUR32 (Ptrif.0004s1277). Homologous genes of *PtrSAUR32* in other species were obtained from the website Phytozome (https://phytozome-next.jgi.doe.gov/) (accessed on 17 January 2025). The software MEGA11 was used to construct a phylogenetic tree by the neighbor-joining method, with a bootstrap of 1000 replicates, and other parameters were in default values. Multiple comparisons of *PtrSAUR32* and its homologous genes were performed using DNAMAN9 software. The website PlantCARE (http://bioinformatics.psb.ugent.be/webtools/plantcare/html/) (accessed on 12 May 2023) was used to analysis the *cis*-acting elements of *PtrSAUR32* promoter (2.5 kb DNA sequence region upstream of ATG).

### 4.5. Subcellular Localization of PtrSAUR32

The *PtrSAUR32* coding sequence, without a termination codon, was fused to the Cam35S-GFP vector. *Agrobacterium* cells containing PtrSAUR32::GFP or Cam35S::GFP were suspended in the infection solution (10 mM MgCl_2_, 10 mM MES, 150 μM acetosyringone, pH 5.6) until the OD_600_ was 1.0. The above suspension was co-injected with *Agrobacterium* cells carrying a red membrane marker into the leaves of a 4-week-old *N. benthamiana*. Fluorescence signals were observed under an FV3000 confocal microscope (Olympus Tokyo, Japan) after infiltration for two days. The primer sequences used for vector construction in this study are listed in Supplementary Data Table S2.

### 4.6. Generation of Transgenic Plants

The full-length CDS of *PtrSAUR32* was subcloned into the pFGC5941MDB3F-GN vector to generate overexpression lines, and a specific DNA fragment of *PtrSAUR32* was subcloned into the pFGC5941MDB3F-GN vector to generate RNA interference (RNAi) lines. The promoter of *PtrSAUR32* was cloned into a p1300GNGM-GUS vector to drive the expression of the GUS reporter gene. The above vectors were transformed into *Agrobacterium tumefaciens* EHA105. Transgenic citrus plants were generated as previously described [52]. Transgenic citrus plants were identified by GFP and genomic PCR.

### 4.7. GUS Staining

The seedlings of WT and the *PtrSAUR32* promoter transgenic plants were used for β-glucuronidase (GUS) staining, and were submerged in GUS dye solution at 37 °C for 12 h and decolorized with 70% ethanol.

### 4.8. Y2H and BiFC Assay

The GAL4 yeast two-hybrid (Y2H) system was used to detect the interactions between PtrSAUR32 and PtrPP2C.Ds, according to Yu et al. [53]. The coding sequence of *PtrSAUR32* was cloned into the GAL4 activation domain vector pGADT7, and coding sequences of *PtrPP2C.Ds* were cloned into the DNA-binding domain vector pGBKT7. Both vectors were co-transformed into the yeast strain Y2H, growing on SD/-Leu/-Trp-deficient medium. The positive colonies were used for selecting interaction proteins on selective SD/-Leu/-Trp/-His/-Ade medium with X-α-gal.

Bimolecular fluorescence complementation (BiFC) assays were performed, as previously described [52]. Full-length coding sequences of *PtrSAUR32* and its candidate interactive proteins were constructed into N-terminal or C-terminal yellow fluorescence protein fragments, respectively, and then co-injected into tobacco leaves. The CDS of PtrPP2C.D7 and PtrHA was constructed into N-terminal and C-terminal yellow fluorescence protein fragments and co-injected into tobacco leaves. The fluorescence signal was observed under a FV3000 confocal microscope.

### 4.9. Transient Expression of PtrARFs

The CDS of *PtrARF1*, *PtrARF5*, *PtrARF6*, *PtrARF7*, *PtrARF8,* and *PtrARF19* were fused into the pFGC5941MDB3F-GN vector, respectively. The fusion vector was introduced into EHA105. *Agrobacterium* cells containing CDS of the target gene were suspended in the infection solution, and citrus leaves were soaked in this solution and vacuumed for 10 min. After three days of infection, the total RNA was extracted for expression analysis.

### 4.10. Y1H and Dual-Luciferase Reporter Assay

For the yeast one-hybrid (Y1H) assay, the *PtrSAUR32* promoter sequence (*proPtrSAUR32*), 2.5 kb upstream region of ATG, was inserted into the vector pHIS2, while the coding sequences of *PtrARF6*, *PtrARF7,* and *PtrARF8* were inserted into pGADT7, respectively. The fused vector pHIS2-PtrSAUR32 was co-transformed into the yeast strain Y187 cell with AD-PtrARF6, AD-PtrARF7, AD-PtrARF8, and pGADT7. The growth of yeast cells on SD/-L-T-H (containing 3-AT) medium was used to determine the interactions between *proPtrSAUR32* and PtrARF6, PtrARF7, and PtrARF8. Yeast cells containing *proPtrSAUR32* and pGADT7 were used as the control.

For the dual-luciferase reporter assay, the promoter of *PtrSAUR32* was cloned into the vector pGreenII 0800-LUC to drive the reporter gene. The CDS of *PtrARF8* was inserted into the vector pGreen II 62-SK to generate the effector construct. The constructed vectors were introduced into GV3101 (pSoup), respectively, and co-injected into *N. benthamiana*. The Dual Luciferase Reporter Gene Assay Kit (Yeasen, Shanghai, China) was used to assess Firefly and Renilla luminescent signals.

## Data Availability

Data are contained within the article and Supplementary Materials.

## Supplementary Materials

The following supporting information can be downloaded at: https://www.mdpi.com/article/10.3390/plants14111579/s1, Figure S1: Phylogenetic relationship and conserved motifs; Figure S2: Multiple sequence alignment of PtrHA with AHA1 and AHA2 of Arabidopsis; Table S1: Primers used for qRT-PCR analysis; Table S2: Primers used for vector construction; Table S3: Root indexes of twelve citrus rootstock varieties at different days after sowing.
