# Supplementary material for: PtrSAUR32 Interacts with PtrPP2C.Ds to Regulate Root Growth in Citrus
Source: Plants (Basel). 2025 May 22;14(11):1579. doi: 10.3390/plants14111579 (PMC12157795; doi:10.3390/plants14111579)
Supplement: Supplementary file 1 [file plants-14-01579-s001.zip › plants-3628079-supplementary/Table S1 Primers used for qRT.pdf]

Table S1 Primers used for qRT-PCR analysis

Gene	Forward primer (5′ to 3′)	Reverse primer (5′ to 3′)	Accession numbers
PtrSAUR44	AGAAGTCGTTTTTCGCTCTCTA	TCTTGGGTCAAGTTAGAGTCAC	Ptrif.0008s0896
PtrSAUR55	AAGGCCACCTAGCAAGATTAAT	AGAAGTGAGGTTAATGAAGGCA	Ptrif.0008s0912
PtrSAUR40	TGTTTCTTGAACGTGGTGTAAC	CTTTCGCGAAAAAGTGATTGTG	Ptrif.0008s0120
PtrSAUR17	AAGAAAGGTTTCCAAGTTTCCG	AGTCCTTGTTGATGATAGCCAA	Ptrif.0004s1277
PtrSAUR34	CCGATGAGAAACGCTTTGTTAT	GCTGCAAATGAATGAATGTTGG	Ptrif.0008s0101
PtrSAUR32	TGGTTTGGGCAATGATACAAAG	ACATGGGATCCTCAAAATTCCT	Ptrif.0007s1022
PtrSAUR38	CCATTTTGTTGTGTACACCACT	GTACTATCACATGGCAGAGTGA	Ptrif.0008s0118
PtrSAUR64	TGTGGTTCCCATTTCTTATT	GATGATTGAAGCCAAACTCT	Ptrif.0008s0919
PtrSAUR18	CACAACAGCATGAGTAACACAA	GAAATTGAAGAGCTAGCTTGGG	Ptrif.0004s2455
PtrSAUR2	CTTAGCAGCCCAAAACCGAG	CGGAACATTTGCAAGCCTCT	Ptrif.0001s1997
PtrSAUR23	GGAAAGATTGGATGTGACGATG	ACATCTCAGCTACAAAACCGTA	Ptrif.0006s0350
PtrSAUR33	CTTAAATCACTTCGTTCGCCAA	GTTTCTTCATGGCTGACTTGAG	Ptrif.0007s1023
PtrSAUR30	CACTTTGTGGTTTATGTGGGAG	GAACCTATATTCCTCAGCTGCT	Ptrif.0007s0796
PtrSAUR11	ACCTATCTTTTGACCATACGCT	ACAGAGTTTTCATTCGAGACCT	Ptrif.0003s4306
PtrSAUR59	CTCACAAGCATCCAAGTCTCTA	TGGCTAGCCTTAAATGAAGACA	Ptrif.0008s0914
PtrSAUR10	GGGGTGTTTGGCGATTAAGG	CATACTCCTCTTCGGCCTCC	Ptrif.0003s3960
PtrSAUR52	AAACTTGCAAAGCTCTAAGACG	GTATGAAACAGGCACCACAAAT	Ptrif.0008s0907
PtrSAUR51	CAACTGTGCATTCTAAACAACG	TTCTTCAGCACTGGGAATTTTG	Ptrif.0008s0901
PtrSAUR8	CAATTGTTGAACGTTCCGAGAT	CAATCTTGAAGTCGAGAAAGCC	Ptrif.0003s3663
PtrSAUR60	TTGTGCTTCCAATCTCCTACTT	ATGATCAAACCCGAACTCTTCT	Ptrif.0008s0915
PtrPP2C.D3	TCCTTCTGCCACCTATGTTGG	ACTTCTGCTGATAATCCACCCTG	Ptrif.0007s0518
PtrPP2C.D1	CCTTCGTTGGTGTTTATGAT	AGCCTTTCTTATAACATTTTCCGACA	Ptrif.0006s0515
PtrPP2C.D7	GAGGACCTGAGACATCGCG	TAGTAACAAGAGACATAAACCCTTCCT	Ptrif.0008s0143
PtrPP2C.D8	CGTGACCATCTTTTTCGCCA	TCCACATGTCCTACGTACAAG	Ptrif.0006s1247
PtrPP2C.D5	CCCGAGGCTTCCAGATTTGT	CCGTACAGGCAATACACGCT	Ptrif.0008s0373
PtrPP2C.D6	ACTCTTGAGTCTGGTCCTTATGG	TGGATTGCTGCTCTGATGTGA	Ptrif.0007s0095
PtrPP2C.D2	CAGAGCCAGATTGAAAGCGG	TGGTTTCTCTAGATACAACTCCCT	Ptrif.0009s0115
PtrPP2C.D4	CTCGATTTGTGAATGACCGCC	ACAACATGACCCAGCAGAGG	Ptrif.0004s0477
PtrARF19	GCAAATGGGTTTCTGCCAAGT	AGGGAAGTAAACAACCAGGCT	Ptrif.0001s2887
PtrARF5	TGGAGGATTGGTTATTCGAGCA	TGAGGGAGGAAAACAAGGGG	Ptrif.0005s2742
PtrARF6	ACGTCGGGGTTTAATCAGCA	AGACAACACGGCTTCCAACA	Ptrif.0002s0903
PtrARF8	GTGTCAACAGGGTCATGAAGG	CTGTTCGCTATGCCCCTGAG	Ptrif.0006s1621
PtrARF1	GGCCCTCTGGTTTCTCTTCC	GGGTAGTTAGGAATATGGGCATCC	Ptrif.0002s2486
PtrARF7	GGAGGAGGGGAGAAGAAGACT	TCTTCATAGATGCTGCCACCT	Ptrif.0001s2230
PtrRR5	CATGCCTGGGATGACTGGAT	ATTCCTCTGCCCCTTCTTCC	Ptrif.0003s0396
PtrRR14	GGTCAGCATCTCAATAGCCTTC	CCATCCTGCTGCTGTTTCAG	Ptrif.0009s0179
PtrPIN1	CAAATGCCTGCCATTGTAGC	TGCAAAGCCATGAACAGACC	Ptrif.0001s1105
PtrPIN3	AATCCCGAGTTTTCGTCTACCC	TGCTGTTGTTGTTGCTGCTG	Ptrif.0002s2006
PtrPIN4	CCGTCCCATTGCTCTCATTTC	TGCCGTGTTTTGTGAAAGCC	Ptrif.0001s2254
PtrPIN3-1	AGCCAGGATGTTGATTGCAG	TCAGCTTCAGTTTGCAACGG	Ptrif.0006s1721
PtrYUC3	GGTTTCTGGCGAATCAAGACC	TGCCTTCGAAATGCTGCAAG	Ptrif.0009s0961
PtrYUC5	AAATGCCGAGCGTGTTATGC	ATGCCAGAATTGCCACAACC	Ptrif.0003s3237
PtrYUC3-1	ATGCGACGATTCGGTTTTGG	ACCGACACAGGTACTCCATTTC	Ptrif.0001s0359
PtrActin	CCGACCGTATGAGCAAGGAAA	TTCCTGTGGACAATGGATGGA	Ptrif.0007s2253
