# Supplementary material for: PtrSAUR32 Interacts with PtrPP2C.Ds to Regulate Root Growth in Citrus
Source: Plants (Basel). 2025 May 22;14(11):1579. doi: 10.3390/plants14111579 (PMC12157795; doi:10.3390/plants14111579)
Supplement: Supplementary file 1 [file plants-14-01579-s001.zip › plants-3628079-supplementary/Table S2 Primers used for vector construction.pdf]

Table S2 Primers used for vector construction

Name	Forward primer (5′ to 3′)	Reverse primer (5′ to 3′)
OE-PtrSAUR32	GCCCAATCGATGATTTAAATATGGGGGTCA	CTCTAGACTCACCTAGGATCCCTAAACTGTTTTA
OE-PtrARF1	GCCCAATCGATGATTTAAATATGAGGCTCTC	CTCTAGACTCACCTAGGATCCTCAGAAATCCAGT
OE-PtrARF5	GCCCAATCGATGATTTAAATATGGGTTCTGT	CTCTAGACTCACCTAGGATCCTCAAGCACGGCC
OE-PtrARF6	GCCCAATCGATGATTTAAATATGAGACTCG	CTCTAGACTCACCTAGGATCCTCAGTAGTTGATA
OE-PtrARF7	GCCCAATCGATGATTTAAATATGAAGACAC	CTCTAGACTCACCTAGGATCCTTAAGCATTCCCA
OE-PtrARF8	GCCCAATCGATGATTTAAATATGAAGCTTTC	CTCTAGACTCACCTAGGATCCTCAATATTCAAGC
OE-PtrARF19	GCCCAATCGATGATTTAAATATGAAGCCTC	CTCTAGACTCACCTAGGATCCTTATCGATTAAAT
RNAi-PtrSAUR32	GCCCAATCGATGATTTAAATTTAAGCTGGTT	AAACCTGACGTCATTTAAATAAACTTGTGATTGG
	TCGTCTTTGTAGTCTCTAGATTAAGCTGGTT	AATGCATGCTTAGGTGGATCCAAACTTGTGATTG
Sub- PtrSAUR32	AGAACACGGGGGACGAGCTCATGGGGGTC	CGACTCTAGAGGATCCAACTGTTTTAACTCTAAA
AD- PtrSAUR32	GTACCAGATTACGCTCATATGATGGGGGTC	ATGCCCACCCGGGTGGAATTCCTAAACTGTTTTA
BK-PtrPP2C.D1	TGGCCATGGAGGCCGAATTCGAAGATGAA	CGCTGCAGGTCGACGGATCCACCACCTCTCATC
BK-PtrPP2C.D2	TGGCCATGGAGGCCGAATTCATGCTCCATG	CGCTGCAGGTCGACGGATCCGTGTTGCAGAGCA
BK-PtrPP2C.D3	TGGCCATGGAGGCCGAATTCATGTTGGAGA	CGCTGCAGGTCGACGGATCCAGCAGAAAGAGA
BK-PtrPP2C.D4	TGGCCATGGAGGCCGAATTCATGGTATCGG	CGCTGCAGGTCGACGGATCCCTATGTGTTGGCA
BK-PtrPP2C.D5	TGGCCATGGAGGCCGAATTCATGATGATGT	CGCTGCAGGTCGACGGATCCTTATGCAGGAAGC
BK-PtrPP2C.D6	TGGCCATGGAGGCCGAATTCCATACGAGTG	CGCTGCAGGTCGACGGATCCAACACCACCCCCT
BK-PtrPP2C.D7	TGGCCATGGAGGCCGAATTCATGTTATCGG	CGCTGCAGGTCGACGGATCCTCAGGTACCACCA
BK-PtrPP2C.D8	TGGCCATGGAGGCCGAATTCATGCTTTCTT	CGCTGCAGGTCGACGGATCCTCAATTTTGTAGAA
YC- PtrSAUR32	GAGCTGTACAAGTCCGGAGTCGACATGGG	GAATTCGAGCTCGCCTGGGGATCCCTAAACTGT
YN- PtrPP2C.D1	AACATCGAGGACTCCGGAGTCGACATGTC	GAATTCGAGCTCGCCTGGGGATCCTCAGTAGTG
YN- PtrPP2C.D6	AACATCGAGGACTCCGGAGTCGACATGTTA	GAATTCGAGCTCGCCTGGGGATCCTTAAGTGGT
YN- PtrPP2C.D7	AACATCGAGGACTCCGGAGTCGACATGTTA	GAATTCGAGCTCGCCTGGGGATCCTCAGGTACC
YC-PtPHA	GAGCTGTACAAGTCCGGAGTCGACATGGA	GAATTCGAGCTCGCCTGGGGATCCTTACACTGTA
AD- PtrARF7	GTACCAGATTACGCTCATATGATGAAGACA	ATGCCCACCCGGGTGGAATTCTTAAGCATTCCCA
AD- PtrARF8	GTACCAGATTACGCTCATATGATGAAGCTTT	ATGCCCACCCGGGTGGAATTCTCAATATTCAAGC
pHIS2-PtrSAUR32	ACGACTCACTATAGGGCGAATTCTACACT	TTCGCGAACGCGTGAGCTCTATTGGCCTCTCTAA
LUC- PtrSAUR32	GGCCCCCCCTCGAGGTCGACTACACTAAA	GCTCTAGAACTAGTGGATCCTATTGGCCTCTCTA
SK- PtrARF8	CCCGGGCTGCAGGAATTCATGAAGCTTTC	TCGACGGTATCGATAAGCTTTCAATATTCAAGC
