# Supplementary material for: PtrSAUR32 Interacts with PtrPP2C.Ds to Regulate Root Growth in Citrus
Source: Plants (Basel). 2025 May 22;14(11):1579. doi: 10.3390/plants14111579 (PMC12157795; doi:10.3390/plants14111579)
Supplement: Supplementary file 1 [file plants-14-01579-s001.zip › plants-3628079-supplementary/Table S3 Root indexes of twelve citrus rootstock v.pdf]

Table S3 Root indexes of twelve citrus rootstock varieties at different days after sowing

Variety	Days after sowing	Primary root length (cm)	Lateral root numbers in RC	Total lateral root numbers
Xiaoyezhi	5	4.80	0	0
	10	10.63	2.67	4
	20	13.20	7.33	11
	30	13.77	8.67	14
Donghu No. 1	5	6.22	0	0
	10	11.20	1.67	2
	20	14.97	3.67	5
	30	15.90	4.00	8
Donghu No. 2	5	4.93	0	0
	10	10.90	2.00	3
	20	13.30	4.33	11
	30	15.83	8.67	11
Bopizhi	5	5.11	0	0
	10	7.93	0.67	1
	20	12.37	6.33	10
	30	12.50	7.33	13
Houpizhi	5	5.31	0	0
	10	10.43	0.67	1.33
	20	13.00	5.00	10
	30	13.70	6.67	10
Tanhezhi	5	6.31	0	0
	10	10.73	3.67	6
	20	12.93	6.67	15
	30	14.03	8.33	17
Volkamer	5	4.27	0	0
	10	9.20	3.67	5
	20	11.57	4.67	10
	30	12.73	5.00	15
Guangxi Tuningmeng	5	4.05	0	0
	10	9.33	1.67	3
	20	11.73	2.00	3.67
	30	12.53	3.67	9
Hongningmeng	5	4.06	0	0
	10	7.47	4.67	4.67
	20	11.17	7.33	11
	30	11.23	7.67	12
Zhecuanzoupigan	5	4.23	0	0
	10	6.03	2.00	3.33
	20	9.60	5.00	10
	30	11.13	6.67	16
Biangan	5	3.89	0	0
	10	6.13	0.67	0.67
	20	9.73	3.67	3.67
	30	9.80	5.00	5
Zhuhongju	5	2.62	0	0
	10	6.47	2.00	3
	20	11.07	4.67	9
	30	11.77	6.00	9
