# Supplementary figures and images for: PtrSAUR32 Interacts with PtrPP2C.Ds to Regulate Root Growth in Citrus
Source: Plants (Basel). 2025 May 22;14(11):1579. doi: 10.3390/plants14111579 (PMC12157795; doi:10.3390/plants14111579)

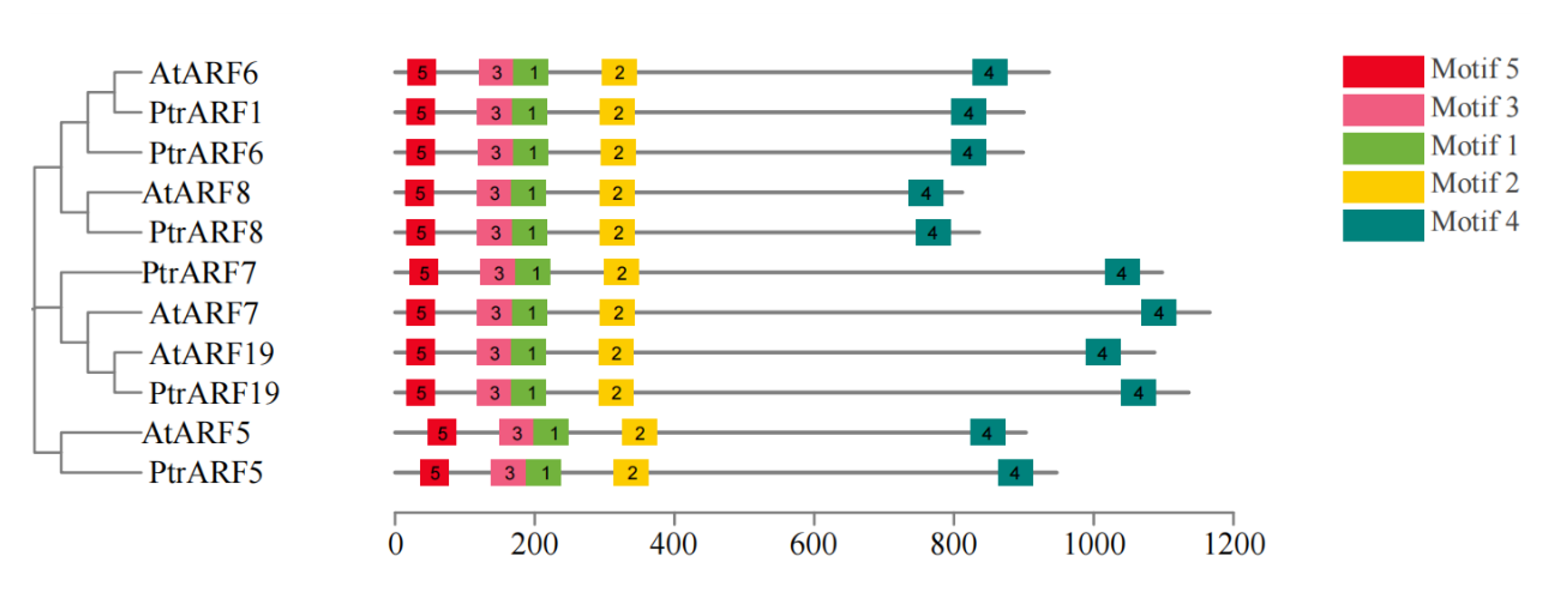

Supplement: Supplementary file 1 [file plants-14-01579-s001.zip › plants-3628079-supplementary/Figure S1.Phylogenetic relationship and conserved motifs .tif]

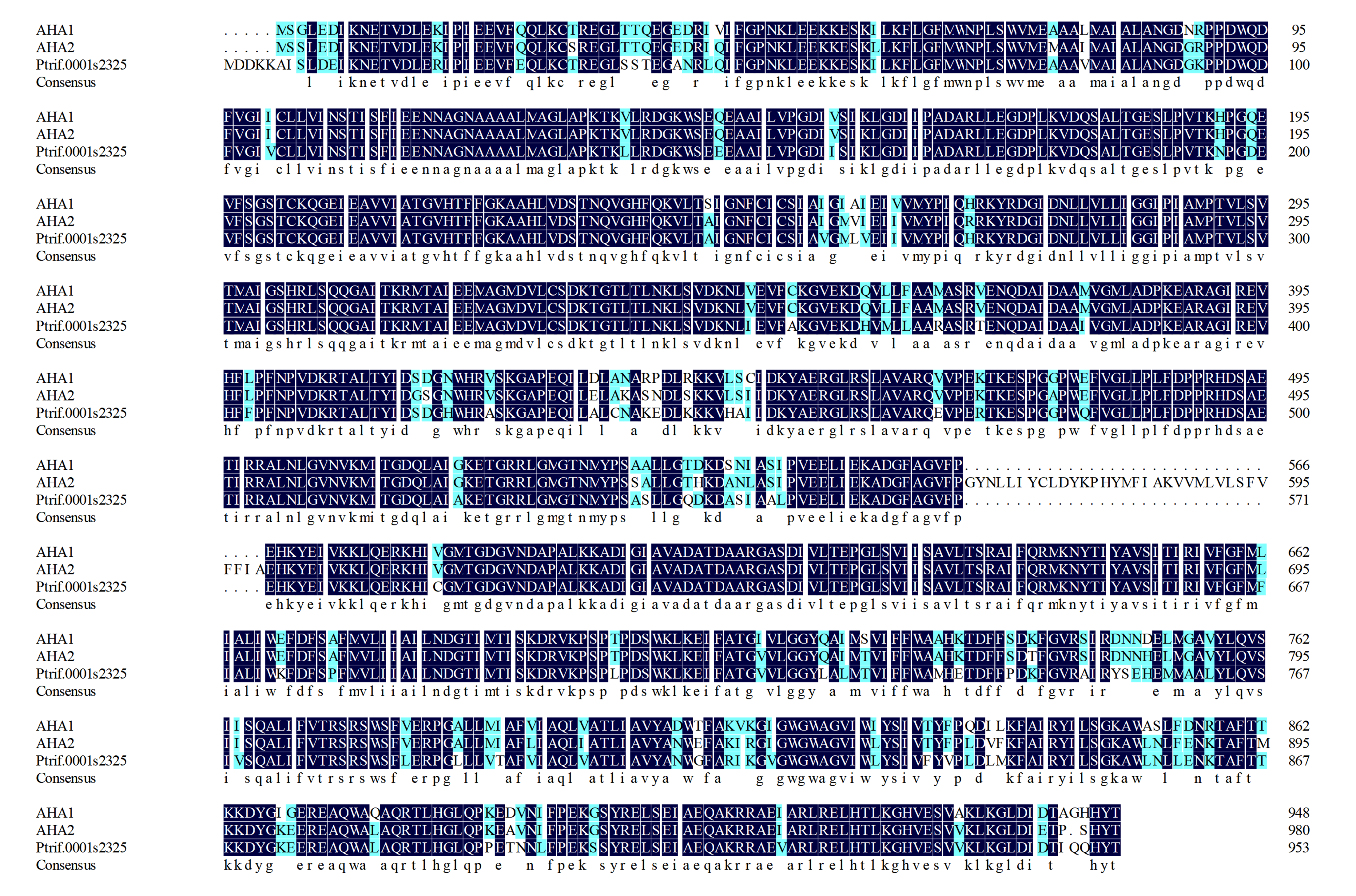

Supplement: Supplementary file 1 [file plants-14-01579-s001.zip › plants-3628079-supplementary/Figure S2 Multiple sequence alignment of PtrHA with AHA1 and AHA2 of Arabidopsis.tif]
